# LEO-Augmented GNSS Based on Communication Navigation Integrated Signal

**DOI:** 10.3390/s19214700

**Published:** 2019-10-29

**Authors:** Lei Wang, Zhicheng Lü, Xiaomei Tang, Ke Zhang, Feixue Wang

**Affiliations:** College of Electronic Science, National University of Defense Technology, Changsha 410073, China; w_lei_81@163.com (L.W.);

**Keywords:** communication and navigation integration, LEO, GNSS, burst signal, IRIDIUM

## Abstract

Low Earth Orbit (LEO) is of great benefit for the positioning performance of Global Navigation Satellite System (GNSS). To realize the system of LEO-augmented GNSS, three methods to integrate communication and navigation signal for LEO communication system with the least influence on the communication performance are analyzed. The analysis adopts the parameters of IRIDIUM signal as restrictions. This paper gives quantitative comparison of these methods considering CN0(carrier noise power spectral density rate) margin, pseudorange accuracy, Doppler accuracy, and communication loss. For method 1, a low-power navigation signal is added to the communication signal. For method 2, the navigation signal is launched in one or more frames. For method 3, the navigation signal is launched in the frequency band separated to the communication signal. The result shows that the pseudorange accuracy of method 2 is far below method 1 and method 3. However, the difference of Doppler accuracy among the three methods can be emitted. Detailed analysis shows that method 1 is practicable when the communication and navigation signal power rate is 15 dB. It achieves the balance of pseudorange accuracy and bit error rate (BER) performance under this condition. Comprehensive comparison of these methods is given in the last. The result shows that the CN0 margin of the navigation signal for method 3 can be 13.04 dB higher than method 1, based on the accuracy threshold considered in this paper. Methods 1 and 3 have the advantage of high accuracy and high CN0 margin respectively. However, method 3 causes high communication capacity loss. Considering that the main disadvantage of GNSS signals is low CN0, method 3 is a good choice for the LEO-augmented GNSS system. Methods 1 and 3 can be combined to realize both high accuracy and high CN0 margin if possible.

## 1. Introduction

A Low Earth Orbit (LEO) constellation has been adopted in satellite communication systems. Considering the high CN0 of the signal received on the ground and high moving velocity of the satellite, GNSS can be augmented by LEO in many aspects. When GNSS is augmented by the LEO satellite system, receivers can realize positioning using the Doppler of signal. Geometric dilution of precision (GDOP) performance can also be improved for positioning with pseudorange. It can also improve the performance of Precise Point Positioning (PPP). Based on these advantages, LEO-augmented GNSS is getting more attention.

There are many LEO satellite communication systems that have been constructed or are under construction, including IRIDIUM [1], GlobalStar [2], Hongyan [3,4], Luojia-1A [5], and so on. GlobalStar is a LEO satellite communication system based on signal transponder. Hongyan and Luojia-1A are still under construction. IRIDIUM is still one of the most important LEO satellite communication systems, and it has developed the iGPS function.

Joerger et al. [6] and Rabinowitz et al. [7] analyzed the performance of LEO-augmented GPS. These analyses are based on the constellation of IRIDIUM, and they show that LEO-augmented GPS shows great improvement in float carrier phase positioning and carrier cycle ambiguity resolution comparing to GPS only. Tian et al. [8] shows generalized analysis of the performance of LEO-augmented GNSS in resolution of integer cycle ambiguities. These papers show that the performance of GNSS can be greatly augmented by LEO satellite system. However, the disadvantage of these analyses is that they are based on simulated measurements. They assume that the pseudorange and carrier phase measurements can be got and they did not consider the signal modulation that LEO satellite system adopts.

Hongyan and Luojia-1A have considered the integration of communication and navigation. However, in the literature, only the performance of navigation signal is provided. The research on integration of communication signal and navigation signal is still not enough. IRIDIUM developed the function of satellite time and location (STL) [9] on the basis of the communication function. The IRIDIUM satellites launch STL burst signals so that receivers could get the pseudorange by measuring the time of arrival (TOA) of the signal. The beginning of a STL burst is manipulated to form a continuous wave (CW) marker, and the remaining in the burst is organized into pseudorandom sequences. But the performance of STL signal is limited under the current design.

There has been some work focusing on the integration of navigation signal and communication signal. He et al. [10] proposes the combination of OFDM and PSK or BOC for the signal modulation of future GNSS. However, this only considers the transmission of navigation messages, and it cannot be used in satellite communication systems. Diez J. et al. [11] developed an integrated navigation and communication system based OFDM modulation. However, this is only for special occasion.

To augment GNSS, the LEO system should supply navigation signal together with communication signal. With the limited bandwidth, the communication signal and navigation signal has to share the bandwidth. Therefore, the integration of the communication signal and navigation signal means the allocation of frequency resource, time resource and power. In this paper the integrated communication signal and navigation signal is considered on the basis of the modulation of IRIDUM. The signal modulation satisfies the satellite communication requirements. The navigation signal is added to the existing communication signal and it is considered with the least influence on the communication system. The following is the quantitative analysis.

### 1.1. Restrictions on Signal Parameters

To give a comprehensive analysis of the integrated navigation and communication signal design, some parameters of the signal have to be restricted, and the IRIDIUM signal system is considered as the basis.

The IRIDIUM signal system is combined of Time Division Multiple Access (TDMA) and Frequency Division Multiple Access (FDMA). The TDMA frame length is 90 ms [12]. The frequency access is 41.667 kHz with the occupied bandwidth being 31.5 KHz. The total bandwidth is 10.5 MHz (1616–1626.5 MHz), and there are 240 duplex frequency access bands and 12 simplex frequency access bands in total.

In the following, the frequency access band will be referred to as unit band with the symbol $B_{u}$, and the occupied bandwidth of every unit band will be expressed as $B_{c}$. The total number of unit band is $N_{B}=252$.The data rate during the data part is 50 Kb/s. The orbit altitude of IRIDIUM is 780 Km.

In this paper, the design of the signal is based on the parameters above. Some other restrictions are set as follows:

(a) Direct Sequence Spread Spectrum (DSSS) and BPSK are considered for the navigation signal.
(b) Assume that the orbit altitude is 24,000 km for medium Earth orbit (MEO). Then, the power deterioration difference for MEO and LEO will be 20 × log(24000/780) = 29.76 dB. This means that when the launch power of the signal is the same, the received power of signal from LEO will be 29.76 dB higher than that from MEO for receivers on the ground. In the following, the CN0 difference is assumed to be 30 dB for simplicity.
(c) The power of each unit band is assumed to be identical, which means that every unit band gets 1/252 of the total power.
(d) The CN0 of GNSS signals mainly falls in 30–50 dBHz in urban areas. Therefore, the analysis assumes that the highest CN0 of the LEO signal is 80 dBHz.

The following analysis is based on the restrictions above.

### 1.2. Methods for Signal Integration

**Figure 1:**
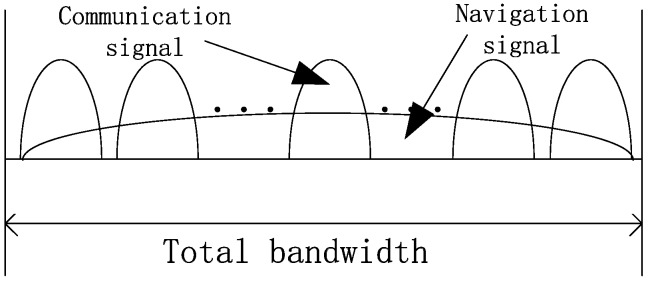
The power spectral density (PSD) of signal generated by method 1.

There are mainly three kinds of methods to realize integration of navigation signal and communication signal. They are described as follows [13,14]:

Method 1: Low-power navigation signal is added to the communication signal. Equation (1) is the expression for method 1 and Figure 1 shows the power spectral density (PSD) of the signal generated by method 1.

(1) $$s\left(t\right)=s_{c}\left(t\right)+s_{nav}\left(t\right)$$

**Figure 2:**
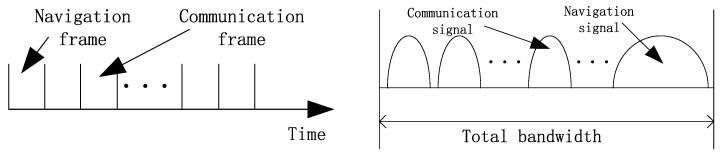
Time domain and PSD of signal generated by method 2.

Method 2: The navigation signal is launched in multiple frames. The expression is shown in panel (2), and Figure 2 shows the time domain and the PSD of the signal generated by method 2.

(2) $$s\left(t\right)=\{\begin{array}{cc} s_{nav}\left(t\right) & t\in t_{nav}\;\&\;f\in f_{nav}\\ s_{c}\left(t\right) & otherwise \end{array}$$

In this paper, the navigation signal is considered to be composed of multiple time frames and multiple unit bands. If the signal is composed of $n_{B}$ unit bands, the bandwidth of the signal is expressed as

(3) $$B_{s}=B_{c}\;+\;\left(n_{B}\;-\;1\right)B_{u}\;1<n_{B}<N_{B}$$

**Figure 3:**
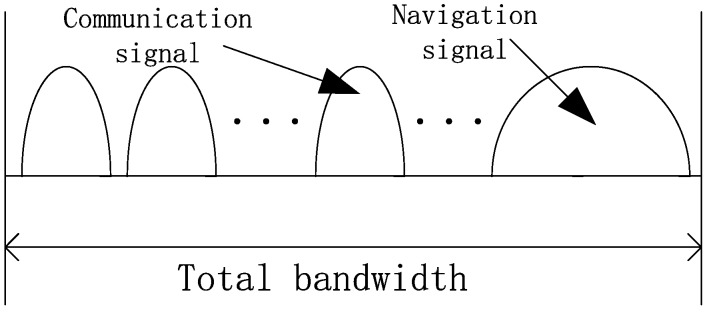
The PSD of signal by generated method 3.

Method 3: The navigation signal is launched in the frequency band separated to the communication signal. The expression is shown in (4) and Figure 3 shows the PSD of signal generated by method 3.

(4) $$s\left(t\right)=\{\begin{array}{cc} s_{nav}\left(t\right) & f\in f_{nav}\\ s_{c}\left(t\right) & otherwise \end{array}$$

The bandwidth of the signal can also be composed of multiple unit bands as Equation (3).

In the concept of STL, the navigation signal is created by method 2. The length of STL signal is 20.32 ms and the bandwidth is one unit band. Limited bandwidth and time length means that the STL signal is of limited performance.

CNSR is used in this paper to describe the communication navigation signal power rate in the following parts. It is expressed as

(5) $$CNSR=P_{c}/P_{nav}$$

where $P_{c}$ is the power of the communication signal and $P_{nav}$ is the power of the navigation signal.

## 2. Performance of the Navigation Signal

Three kinds of measurements can be used for positioning: Doppler, pseudorange, and carrier phase. If the carrier phase is required for positioning, the signal must be continuous. Burst signal can only supply Doppler and pseudorange measurements.

If the signal is continuous, delay lock loop (DLL) can be used to get the pseudorange measurement [15], and frequency lock loop (FLL) can be used to get the Doppler measurement. If the signal is burst, which is true for method 2, only open loop estimation is possible for the corresponding measurements including pseudorange and Doppler.

### 2.1. Pseudorange Accuracy

The accuracy of DLL for methods 1 and 3 and the accuracy of the open loop estimation for method 2 are compared to evaluate the accuracy of pseudorange.

The pseudorange accuracy of open loop estimation based on the noncoherent early minus later power discriminator is shown below [16].

(6) $$\sigma_{OL}=\sqrt{\frac{\tau_{b}}{2C/N_{0}\cdot T_{c\mathrm{oh}}}\left(1+\frac{1}{C/N_{0}\cdot T_{c\mathrm{oh}}\left(1-\tau_{b}\right)}\right)}\left(chips\right)$$

where $\tau_{b}$ is the search interval, $T_{coh}$ is the coherent integration time, and $C/N_{0}$ is the CN0 of the navigation signal.

The pseudorange accuracy caused by thermal noise for DLL based on the noncoherent early minus later power discriminator is shown below when the correlation interval D is set to 0.5 [14].

(7) $$\sigma_{DLL}=\sqrt{\frac{B_{L}}{2\cdot C/N_{0}}\frac{1}{B_{fe}T_{c}}\left(1+\frac{1}{T_{coh}C/N_{0}}\right)}\left(chips\right)$$

where $B_{L}$ is the loop noise bandwidth and it is set as 1 Hz in this paper, $B_{fe}$ is the radio frequency bandwidth, and $T_{c}$ is the chip width. In this paper, $B_{fe}$ is assumed to be $2f_{c}$, and $f_{c}$ is the code rate.

Considering that, for burst signal, the receiver does not have to deal with the signal in real-time, the correlation interval can be lower and $T_{coh}$ can be longer. Therefore, $\tau_{b}$ is set as 0.1, which is lower than the correlation interval D of DLL.

In the following the pseudorange accuracy of open loop estimation and DLL are compared. The following parameters are set to get the comparison result.

$\sigma_{OL}^{1}$: $B_{nav}=31.5\;\mathrm{KHz}$, $T_{coh}=1\;\mathrm{ms}$
$\sigma_{OL}^{2}$: $B_{nav}=31.5\;\mathrm{KHz}$, $T_{coh}=90\;\mathrm{ms}$
$\sigma_{\mathrm{DLL}}^{1}$: $B_{nav}=10.5\;M\mathrm{Hz}$, $T_{coh}=1\;\mathrm{ms}$
$\sigma_{\mathrm{DLL}}^{2}$: $B_{nav}=31.5\;\mathrm{KHz}$, $T_{coh}=1\;\mathrm{ms}$

$\sigma_{OL}^{1}$ and $\sigma_{OL}^{2}$ correspond to method 2, $\sigma_{\mathrm{DLL}}^{1}$ corresponds to method 1, and $\sigma_{\mathrm{DLL}}^{2}$ corresponds to method 3. The following figure shows the comparison results.

**Figure 4:**
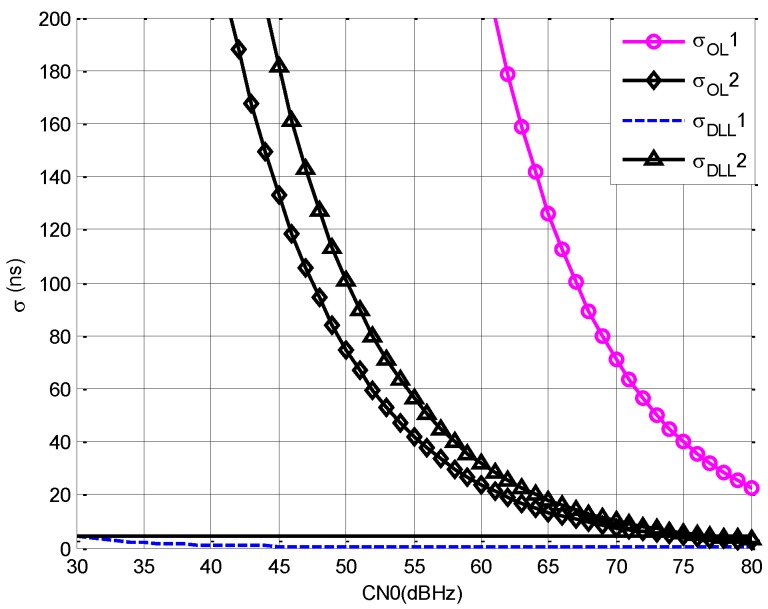
The accuracy of pseudorange versus carrier noise power spectral density rate (CN0).

From Figure 4, we draw the following conclusions.

(1) Comparing $\sigma_{OL}^{1}$ with $\sigma_{OL}^{2}$ and $\sigma_{\mathrm{DLL}}^{2}$, it can be seen that with the increment of $T_{coh}$, σ decreases for open loop estimation. However, the advantage of open loop estimation over DLL is low when the bandwidth is the same.
(2) Comparing $\sigma_{OL}^{1}$ with $\sigma_{\mathrm{DLL}}^{2}$, it can be seen that the accuracy of DLL is better than open loop estimation even though $\tau_{b}$ is lower than D.
(3) Comparing $\sigma_{\mathrm{DLL}}^{1}$ with $\sigma_{\mathrm{DLL}}^{2}$, it can be seen that the increment of $B_{nav}$ causes great decrement of σ, and, when CN0 is 77 dBHz, $\sigma_{\mathrm{DLL}}^{2}$ is about the same with $\sigma_{\mathrm{DLL}}^{1}$ when CN0 is 30 dBHz.

Based on the analysis above, it can be concluded that, the accuracy of method 2 is far worse than methods 1 and 3, and $B_{nav}$ should be increased to get higher accuracy for all methods.

### 2.2. Doppler Accuracy

The accuracy of DLL for methods 1 and 3 and the accuracy of the open loop estimation for method 2 are compared to evaluate the accuracy of Doppler.

The accuracy of open loop estimation for frequency based on differential power discriminator is shown below [17,18].

(8) $$\begin{array}{l} \sigma_{f}=\sqrt{\frac{\mu_{0}}{4\cdot T_{coh}C/N_{0}}\left(1+\frac{\mu_{1}}{T_{coh}C/N_{0}}\right)}\\ \mu_{0}=\frac{f_{b}^{2}\left(1-\cos\left(2\pi f_{b}T_{coh}\right)\right)}{\left(\sin c\left(\pi f_{b}T_{coh}\right)\right)-\cos{\left(\pi f_{b}T_{coh}\right)}^{2}}\\ \mu_{1}=\frac{1-\sin c^{2}\left(2\pi f_{b}T_{coh}\right)}{2\left(1-\cos\left(2\pi f_{b}T_{coh}\right)\right)\sin c^{2}\left(\pi f_{b}T_{coh}\right)} \end{array}$$

where $f_{b}$ is the search interval of frequency and its value should satisfy $f_{b}<\frac{1}{2T_{coh}}$. C/N_0_ is the CN0 of the navigation signal.

The accuracy of FLL for continuous signal is shown as follows [14],

(9) $$\sigma_{FLL}=\frac{1}{2\pi T_{coh}}\sqrt{\frac{4FB_{L}}{C/N_{0}}\left(1+\frac{1}{T_{coh}\cdot C/N_{0}}\right)}$$

F is a parameter, and it is set to 1 in this paper. $B_{L}$ is the loop noise bandwidth, and it is set to 2 Hz.

From Equations (8) and (9), it can be seen that the accuracy of Doppler is mainly determined by $T_{coh}$ and CN0. Another important parameter for accuracy of open loop estimation is $f_{b}$. Therefore, the effects of $f_{b}$ are also considered in the following analysis.

In the following, the Doppler accuracy of open loop estimation and FLL are compared. The following parameters are set to get the comparison result,

$\sigma_{OL}^{1}$: $f_{b}=30,T_{coh}=1\;\mathrm{ms}$$\sigma_{OL}^{3}$: $f_{b}=40,T_{coh}=10\;\mathrm{ms}$
$\sigma_{OL}^{2}$: $f_{b}=30,T_{coh}=10\;\mathrm{ms}$$\sigma_{FLL}$: $T_{coh}=1\;\mathrm{ms}$

$\sigma_{OL}^{1}$, $\sigma_{OL}^{2}$, and $\sigma_{OL}^{3}$ correspond to method 2, and $\sigma_{FLL}$ corresponds to methods 1 and 3. The comparison result is shown below.

**Figure 5:**
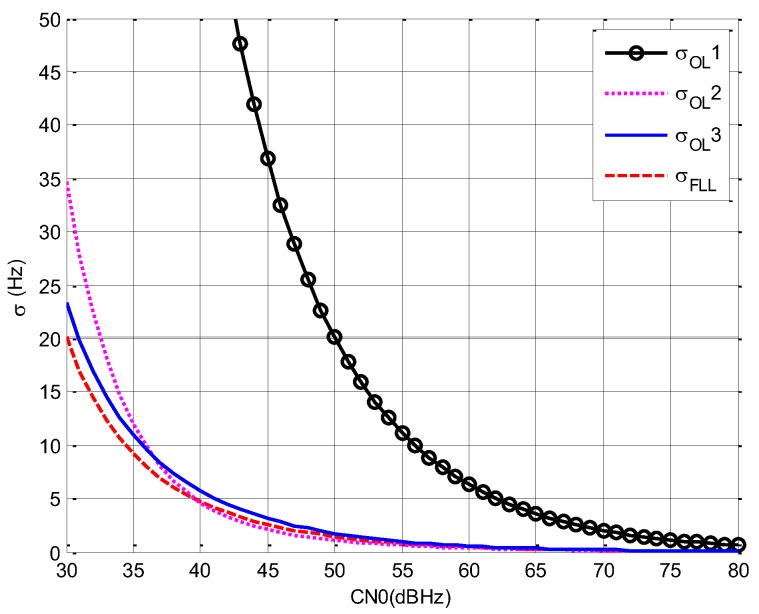
The accuracy of Doppler versus CN0.

From Figure 5, we draw the following conclusions.

(1) Comparing $\sigma_{OL}^{1}$ with $\sigma_{OL}^{2}$, it can be seen that with the increment of $T_{coh}$, σ decreases greatly.
(2) Comparing $\sigma_{OL}^{2}$ with $\sigma_{OL}^{3}$, it can be seen that the effect of $f_{b}$ to σ is not the same during the whole CN0 range. When CN0 is lower than 37 dBHz, $f_{b}=40$ shows better performance, and when CN0 is higher than 37 dBHz, $f_{b}=30$ shows better performance. The optimal choice of $f_{b}$ is another optimization objective and it is not analyzed in this paper.
(3) Comparing $\sigma_{FLL}$ with the others, it can be seen that when $T_{coh}$ is the same, FLL is better than open loop estimation and, if $T_{coh}$ is increased, the accuracy of open loop estimation is close to FLL.

Based on the analysis above, it can be concluded that for method 1 and method 3, the accuracy of Doppler depends only on CN0 of the navigation signal, and for method 2, the accuracy of Doppler can be close to method 1 and method 3 when the receiving parameters are chosen optimally.

Therefore, the difference of Doppler accuracy among the three methods will be emitted in the following.

### 2.3. CN0 of the Navigation Signal

For methods 2 and 3, the power of navigation signal is assumed to be identical to the total power of the communication signal in the same band. Therefore, the CN0 of the navigation signal is shown as follows,

(10) $$CN0_{nav}=CN0_{total}+10\log\left(n_{B}/N_{B}\right)$$

where $CN0_{total}$ is the rate of the total power of the received signal and $N_{0}$. It is expressed as follows,

(11) $$CN0_{total}=\frac{P_{c}+P_{nav}}{N_{0}}$$

It should be noted that if the band of navigation signal and communication signal are separated, navigation signal and communication signal will not interfere each other. Therefore, the power of the navigation signal can be increased according to the requirements for methods 2 and 3.

For method 1, the communication signal is high power jamming for the navigation signal. Note that the communication signal is composed of many unit bands with the same power and the bandwidth of navigation signal is times of the unit band. Therefore, the communication signal can be treated as white noise and the power is the same as the power in the total band. The PSD of the effective white noise should be $P_{c}/B$, where B is the total bandwidth. Then, the effective CN0 of navigation signal for the receiver is

(12) $$\mathrm{CN}0_{eff}=\frac{P_{nav}}{P_{c}/B+N_{0}}$$

The following figure shows the effective CN0 of the navigation signal versus $CN0_{total}$ under different value of CNSR.

**Figure 6:**
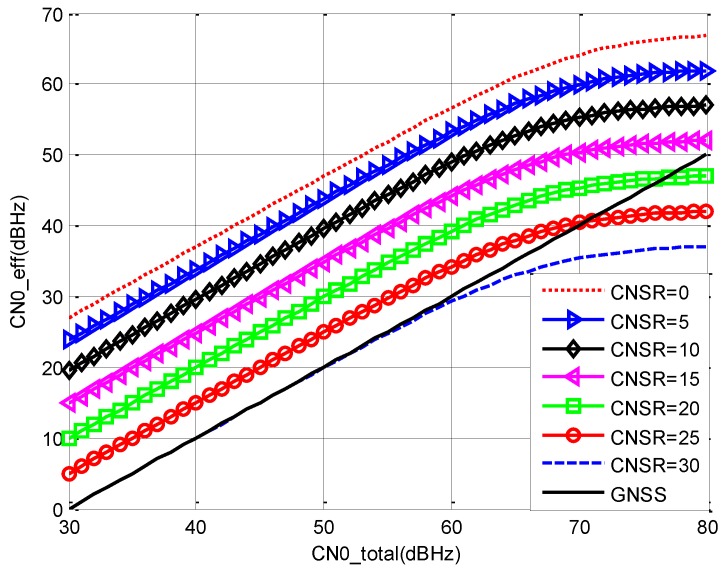
The $\mathrm{CN}0_{eff}$ versus $CN0_{total}$ under different CNSR.

In Figure 6, the legend means CNSR in dB and the black full line is the corresponding CN0 of GNSS signals. From Figure 6, we draw the following conclusions.

(a) When $CN0_{total}$ is below 60 dBHz, $\mathrm{CN}0_{eff}$ increases almost linearly with the increment of $CN0_{total}$. When $CN0_{total}$ is above 70 dBHz, the effective PSD of the communication signal grows to be larger than thermal noise. Therefore, the increment rate of CN0_eff_ goes down.
(b) When $CN0_{total}$ is fixed, $\mathrm{CN}0_{eff}$ decreases with the increment of CNSR. When CNSR is lower than 15 dB, $CN0_{eff}$ holds advantage over the CN0 of GNSS signals for the whole range. Therefore, the value of CNSR should be lower than 15 dB.

## 3. Performance of Communication Signal

The influence of navigation signal on communication signal mainly contains two aspects: the BER performance and the communication capacity loss.

For method 1, the navigation signal becomes interference to the communication signal, and it decreases the BER performance. Therefore, the power of the navigation signal should be limited to guarantee the BER requirement. Method 1 does not cause any capacity loss.

For methods 2 and 3, the bands of navigation signal and communication signal are separated. The power of navigation signal would not affect the BER performance of the communication signal. However, the cost is communication capacity loss.

### 3.1. BER Performance

To obtain the limit of the navigation signal, BER is analyzed. The procedure when receiving the communication signal is as follows. First, the signal is filtered by a band limited filter. Second, the signal is down frequency converted to zero frequency with the locally generated carrier. Third, the signal is integrated during the bit length $\left[0,T_{b}\right]$ to improve the SNR. During this process, the navigation signal in the bandwidth will also be integrated, and the power becomes jamming for the communication signal.

The signal expression after the filter is shown below.

(13) $$y\left(t\right)=h\left(t\right)\left(S_{c}\left(t\right)+S_{nav}\left(t\right)+n\left(t\right)\right)$$

where h(t) is the expression of the filter and the expression of $S_{c}\left(t\right)$, shown below.

(14) $$S_{c}\left(t\right)=\sqrt{2P_{c}}b\left(t\right)\cos\left(2\pi \left(f_{0}+f_{d}\right)t+\phi_{0}\right)$$

where $f_{0}$ is the default frequency of the signal, $f_{d}$ is the Doppler frequency, b(t) is the communication bit, $\phi_{0}$ is the initial carrier phase, and $P_{c}$ is the power of the communication signal.

After obtaining a frequency converter with local generated carrier and integration, the signal is shown below.

(15) $$\begin{array}{cc} Y^{\prime } & =\int_{t=0}^{T_{b}}y\left(t\right)\sqrt{2}\cos\left(2\pi \left(f_{0}+f_{d}\right)t+\phi_{0}\right)dt\\ & =\int_{t=0}^{T_{b}}\left(S_{c}\left(t\right)+h\left(t\right)S_{nav}\left(t\right)+n\left(t\right)\right)\sqrt{2}\cos\left(2\pi \left(f_{0}+f_{d}\right)t+\phi_{0}\right)dt\\ & =\sqrt{P_{c}}b_{0}\left(0\right)T_{b}+\sqrt{P_{nav}^{\prime }}c_{nav}^{\prime }T_{b}+\int_{0}^{T_{b}}\sqrt{2}n\left(t\right)\cos\left(2\pi \left(f_{0}+f_{d}\right)t+\phi_{0}\right)dt \end{array}$$

where $P_{nav}^{\prime }$ is the power of the navigation signal after integration and $c_{nav}^{\prime }$ is the sign. Note that to get the worst BER performance, it is assumed, in (15), that the navigation signal will become a signal of constant power with the sign being positive or negative after the filter and integration. $P_{nav}^{\prime }$ is equal to the accumulated power in the occupied bandwidth of the communication signal and the highest value is obtained when the communication band locates at the central of the PSD of the navigation signal. Under this condition, $P_{nav}^{\prime }$ is expressed as

(16) $$P_{nav}^{\prime }=P_{nav}\frac{\int_{-B_{c}/2}^{B_{c}/2}G_{nav}\left(f\right)df}{\int_{-B_{nav}/2}^{B_{nav}/2}G_{nav}\left(f\right)df}$$

where $B_{c}$ is the occupied bandwidth of the communication signal,$B_{nav}$ is the bandwidth of the navigation signal, $G_{nav}\left(f\right)$ is the PSD of the navigation signal, and $P_{nav}$ is the power of the navigation signal. After integration, the noise is $\Lambda \sim Ν\left(0,N_{0}T_{b}/2\right)$.

From Equation (16), it can be seen that, when the CNSR is the same, the increment of the bandwidth of the navigation signal decreases the value of $P_{nav}^{\prime }$. Therefore, it would be an optimized option when the bandwidth of the navigation signal is as large as possible.

Based on the analysis above, the BER given in [19] is as follows,

(17) $$P_{e}=P_{e1}+P_{e2}+P_{e3}+P_{e4}$$

(18) $$\begin{array}{cc} P_{e1}=P_{e4} & =P_{r}\left\{\Lambda <\left(-\sqrt{P_{c}}T_{b}-\sqrt{P_{nav}^{\prime }}T_{b}\right)\right\}\\ & =Q\left(\sqrt{\frac{2E_{b}\left(1+\sqrt{P_{nav}^{\prime }/P_{c}}\right)}{N_{0}}}\right) \end{array}$$

(19) $$\begin{array}{cc} P_{e2}=P_{e3} & =P_{r}\left\{\Lambda <\left(\sqrt{P_{c}}T_{b}-\sqrt{P_{nav}^{\prime }}T_{b}\right)\right\}\\ & =Q\left(\sqrt{\frac{2E_{b}\left(1-\sqrt{P_{nav}^{\prime }/P_{c}}\right)}{N_{0}}}\right) \end{array}$$

Considering that the data rate is 50 Kb/s, the BER performance of one unit band versus $CN0_{total}$ is shown below when the bandwidth of the navigation signal is 10.5 MHz. This is the worst BER performance among all the communication bands.

**Figure 7:**
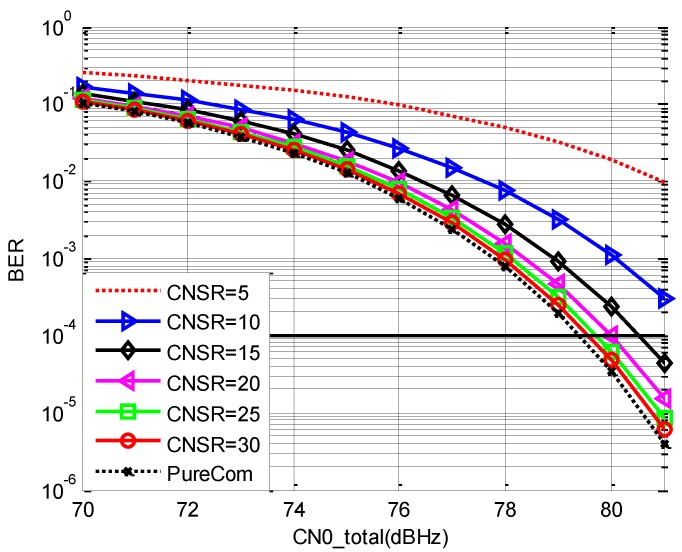
Bit error rate (BER) under versus $CN0_{total}$ under different CNSR.

The legend of Figure 7 is the value of CNSR in dB, and ‘PureCom’ means that there is no navigation signal. The BER threshold is considered as 10^−4^ for satellite communication, and it is shown in the figure with black full line. From Figure 7, when $CN0_{total}$ is fixed, BER performance increases with the increment of CNSR. When CNSR is above 15, $CN0_{total}$ should be increased less than 1 dB to maintain the BER performance.

According to Section 2.3, CNSR should be as low as possible so that $\mathrm{CN}0_{eff}$ of the navigation signal is high. According to the BER performance, CNSR should be as high as possible. Therefore, the best choice for CNSR when the bandwidth of the navigation signal is 10.5 MHz should be the balance of the communication performance and navigation performance. Based on the analysis above, 15 dB is the optimal choice for CNSR.

### 3.2. Communication Capacity

When the navigation signal does not exist, the whole communication capacity is expressed as

(20) $$T_{total}=T_{c}N_{c}$$

where $T_{c}$ is the number of frames in one unit band during the time length T and $N_{c}$ is the number of unit bands.

For method 2, the capacity loss of the communication signal is determined by the number of frames and the unit bands that the navigation has occupied. It is expressed as follows,

(21) $$V_{loss}=\frac{T_{N}N_{N}}{T_{total}}$$

where $T_{N}$ is the number of frames that navigation signal has occupied during the time length T and $N_{N}$ is the number of occupied unit bands.

For method 3, the capacity loss is determined by the number of unit bands that the navigation signal has occupied, which is shown as follows,

(22) $$V_{loss}=\frac{N_{N}}{N_{c}}$$

## 4. Comprehensive Comparison

To get the comprehensive comparison of different methods, two combined aspects are considered as the criterions: CN0 margin and pseudorange precision. CN0 margin is the difference between the CN0 of the navigation signal in the integrated signal system and the normal GNSS signal.

The lowest accuracy threshold is set as 33 ns (10 m) when the CN0 is 40 dBHz and $T_{coh}$ is 10 ms. The performance of different methods is analyzed when the lowest accuracy threshold is satisfied.

### 4.1. Comparison of Methods 1 and 3

To satisfy the accuracy requirement above, the bandwidth of the signal for method 3 can be obtained through Equation (7). The σ calculated in chips is 0.005. Therefore, the code rate should be larger than σ/33 ns, which is 0.1523 MHz; it is ~7.3 times that of the unit band. Therefore, the bandwidth is set as 323.19 KHz, which is equal to eight unit bands. The capacity loss of the navigation signal is 3.17%. The highest CN0 of the navigation signal is 64.84 dBHz, calculated by Equation (10).

From Figure 4, it can be seen that the accuracy threshold can be satisfied by the signal of method 1.

According to Section 2.3 and Section 3.1, CNSR is set as 15 dB for method 1. The CN0 margin of methods 1 and 3 is shown below, and the corresponding pseudorange accuracy is also shown below.

**Figure 8:**
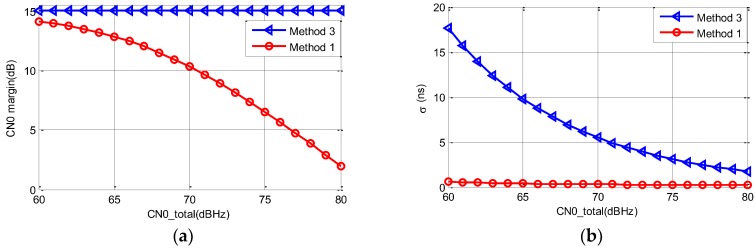
(**a**) CN0 margin versus $CN0_{total}$ for methods 1 and 3. (**b**) The accuracy of pseudorange versus $CN0_{total}$.

From Figure 8, it can be seen that the CN0 margin of method 3 does not change with the increment of $CN0_{total}$, but the CN0 margin of method 1 decreases significantly. The highest CN0 margin of the navigation signal is about 14 dB for method 1. The CN0 margin of method 3 can be 13.04 dB higher than method 1 when $CN0_{total}$ is 80 dBHz.

It can also be seen that the pseudorange accuracy of method 1 is still better than method 3, even though the CN0 margin is lower.

Considering that $CN0_{total}$ can be increased, the CN0 margin can be improved further. For method 3, when the CN0 of the navigation signal is increased to 80 dBHz and the power of the communication signal is kept the same, $CN0_{total}$ increases to 82.94 dBHz. The CN0 margin becomes 30 dB for method 3. At this time, the CN0 margin increment for method 1 is ~2.08 dB, as calculated by Equation (12).

It can be concluded that when the accuracy satisfies the requirement, method 3 keeps the advantage in CN0 margin and the increment of CN0 margin is easy to realize. This can solve the problem of signal weakness in urban valley or indoor environment. Hence it can be a supplement to GNSS.

### 4.2. Comparison of Methods 2 and 3

The signal for method 2 is also composed of multiple unit bands. Therefore, it also holds the advantage of CN0 margin. As the receiver does not have to deal with the signal data in real-time for burst signal, it should satisfy the accuracy requirement when CN0 is 40 dBHz, $T_{coh}$ is 90 ms, and $\tau_{b}$ is 0.1. The result of σ is 0.0075, which is calculated from Equation (6) based on the parameters set above. Therefore, the code frequency should be 0.226 MHz, which means that the number of occupied unit bands would be 11 (0.4482 MHz).

When the occupied navigation frame is 1 out of 10 frames, the capacity loss is 0.44%. The capacity loss of method 3 in 3.2 is 3.17%, which is about 7.27 times of method 2.

It can be concluded that method 2 causes really low communication capacity loss. But to satisfy the accuracy threshold, the receiver has to increase $T_{coh}$ and decrease $\tau_{b}$.

### 4.3. Summary

Based on the analysis above, the characteristics of the three methods can be summarized as the following Table 1.

Method 1 has high accuracy, and it holds a medium CN0 margin against the GNSS signal based on the CNSR chosen in this paper.

Considering that positioning based on Doppler can only be used for static targets and the low pseudorange precision, method 2 is not a good choice for navigation.

Method 3 is of medium accuracy and high CN0 margin. The communication loss is also acceptable. This would be great advantage in the environment when the GNSS signal is deteriorated severely. The accuracy can be improved further with the improvement of receiving algorithm and the usage of carrier phase.

Considering that the great weakness of GNSS signal is low CN0, method 3 would be a good choice of LEO for GNSS augmentation.

## 5. Conclusions

Three methods for integration of navigation signal and communication signal with the least influence on the communication performance are analyzed comprehensively. The pseudorange accuracy of method 1 is high, but it causes interference to the communication signal. Our analysis shows that the balance of navigation performance and communication performance is achieved when CNSR is 15 dB. Under this condition, the navigation signal is of high effective CN0 and the increment of $CN0_{total}$ to maintain the BER performance is less than 1dB. The accuracy of method 2 is far lower than the others. The Doppler accuracy of the three methods is identical, but the integration time length of method 2 is much higher than methods 1 and 3.

Comparing method 1 with method 3, it can be got that method 1 is of higher pseudorange accuracy. However, the CN0 margin of method 3 can be up to 13.04 dB higher than method 1. The accuracy and communication capacity loss are analyzed for methods 2 and 3. The capacity loss of method 3 is 7.27 times of method 2, but method 3 holds the advantage of high pseudorange accuracy. The performance can be improved further with the usage of carrier phase for method 3 compared with method 2. To conclude, method 3 can reach the balance of accuracy and CN0 margin to realize the LEO-augmented GNSS system. Methods 1 and 3 can be combined if possible.

## Figures and Tables

**Table 1:** Summary of the characteristics of three methods.

	Method 1	Method 2	Method 3
Pseudorange precision	High	Low	Medium
Doppler precision	High	Medium	High
CN0 margin	Medium	High	High
Positioning method	Pseudorange/Doppler/Carrier phase	Pseudorange/Doppler	Pseudorange/Doppler/Carrier phase
Communication loss	Low	Low	Medium

## References

[1] Iridium NEXT: A Global Effort to Launch the Future of Global Communications. http://www.iridium.com.

[2] Zhang H., Wang M.C., Cui W.Z. (2018). Satellite Comunication.

[3] Meng Y., Bian L., Han L., Lei W., He M. A Global Navigation Augmentation System Based on LEO Communication Constellation. Proceedings of the European Navigation Conference.

[4] Meng Y., Bian L., Han L., Lei W., Yan T., He M., Li X. (2019). A global navigation augmentation system based on LEO communication constellation. J. Terahertz Sci. Electron. Inf. Technol..

[5] Wang L., Chen R., Li D., Zhang G., Shen X., Yu B., Wu C., Xie S., Zhang P., Li M. (2019). Initial Assessment of the LEO Based Navigation Signal Augmentation System from Luojia-1A Satellite. Sensors.

[6] Joerger M., Gratton L., Pervan B., Cohen C.E. (2010). Analysis of Iridium-augmented GPS for floating carrier phase positioning. Navigation.

[7] Rabinowitz M., Parksinson B.W., Gromov K. Architectures for Joint GPS/LEO Satellite Carrier Phase Receivers Designed for Rapid Robust Resolution of Carrier Cycle Ambiguities on Mobile Platforms. Proceedings of the 13th International Technical Meeting of the Satellite Division of The Institute of Navigation.

[8] Tian S., Dai W., Liu R., Chang J., Li G. (2014). System Using Hybrid LEO-GPS Satellites for Rapid Resolution of Integer Cycle Ambiguities. IEEE Trans. Aerosp. Electron. Syst..

[9] David W.B. (2010). iGPS: Integrated Nav & Com Augmentation of GPS.

[10] He T., Ma Z. (2016). Proposed OFDM Modulation for Future Generations of GNSS Signal System. J. Navig..

[11] Diez J., de Castro D., Palomo J.M., Tossaint M. Integrated Navigation and Communication System based on OFDM. Proceedings of the 5th ESA Workshop on Satellite Navigation Technologies and European Workshop on GNSS Signals and Signal Processing.

[12] Pratt S.R., Raines R.A., Fossa C.E., Temple M.A. (1999). An Operational and Performance Overview of the Iridium Low Earth Orbit Satellite System. IEEE Commun. Surv..

[13] Huang X., Zhao X., Zhu X., Ou G. MC-BOC: A New Interoperable Modulation and Performance Analysis for BeiDou B1 Signal. Proceedings of the China Satellite Navigation Conference (CSNC).

[14] Deng Z., Yuan X., Yu Y. (2013). A Novel Pseudo Code Ranging Method for High Accurate Cellular Positioning Receiver. Adv. Inf. Sci. Serv. Sci..

[15] Elliott D., Hegarty C. (2012). Understanding GPS: Principles and Applications.

[16] Ruan H., Li J., Zhang L., Long T. (2015). Adaptive Correlation Space Adjusted Open Loop Tracking Approach for Vehicle Positioning with Global Navigation Satellite System in Urban Areas. Sensors.

[17] Tang X., Falletti E., Presti L. Fine Doppler Frequency Estimation in GNSS Signal Acquisition Process. Proceedings of the 6th ESA Workshop on NAVITEC.

[18] Lin H., Tang X., Ou G. (2017). An Open Loop with Kalman Filter for Intermittent GNSS Signal Tracking. IEEE Commun. Lett..

[19] Hu Y., Song M.Z., Dang X.Y. (2015). A Method for the Navigation Satellite Signal Enhancement Based on the Signal Retransmission by the Communication Satellite. J. Electron. Inf. Technol..

